# Promoter-Level Transcriptome Identifies Stemness Associated With Relatively High Proliferation in Pancreatic Cancer Cells

**DOI:** 10.3389/fonc.2020.00316

**Published:** 2020-03-20

**Authors:** Ru Chen, Aiko Sugiyama, Naoyuki Kataoka, Masahiro Sugimoto, Shoko Yokoyama, Akihisa Fukuda, Shigeo Takaishi, Hiroshi Seno

**Affiliations:** ^1^Department of Gastroenterology and Hepatology, Graduate School of Medicine, Kyoto University, Kyoto, Japan; ^2^DSK Project, Medical Innovation Center, Graduate School of Medicine, Kyoto University, Kyoto, Japan; ^3^Laboratory of Cell Regulation, Department of Applied Animal Sciences, Graduate School of Agricultural and Life Sciences, The University of Tokyo, Tokyo, Japan; ^4^Department of Applied Biological Chemistry, Graduate School of Agricultural and Life Sciences, The University of Tokyo, Tokyo, Japan; ^5^Research and Development Center for Minimally Invasive Therapies Health Promotion and Preemptive Medicine, Tokyo Medical University, Tokyo, Japan

**Keywords:** cap analysis of gene expression, intraductal papillary mucinous neoplasm, molecular profiling, pancreatic intraepithelial neoplasia, transcriptome

## Abstract

Both pancreatic intraepithelial neoplasia (PanIN), a frequent precursor of pancreatic cancer, and intraductal papillary mucinous neoplasm (IPMN), a less common precursor, undergo several phases of molecular conversions and finally develop into highly malignant solid tumors with negative effects on the quality of life. We approached this long-standing issue by examining the following PanIN/IPMN cell lines derived from mouse models of pancreatic cancer: Ptf1a-Cre; Kras^G12D^; p53^f/+^ and Ptf1a-Cre; Kras^G12D^; and Brg1^f/f^ pancreatic ductal adenocarcinomas (PDAs). The mRNA from these cells was subjected to a cap analysis of gene expression (CAGE) to map the transcription starting sites and quantify the expression of promoters across the genome. Two RNA samples extracted from three individual subcutaneous tumors generated by the transplantation of PanIN or IPMN cancer cell lines were used to generate libraries and Illumina Seq, with four RNA samples in total, to depict discrete transcriptional network between IPMN and PanIN. Moreover, in IPMN cells, the transcriptome tended to be enriched for suppressive and inhibitory biological processes. In contrast, the transcriptome of PanIN cells exhibited properties of stemness. Notably, the proliferation capacity of the latter cells in culture was only minimally constrained by well-known chemotherapy drugs such as GSK690693 and gemcitabine. The various transcriptional factor network systems detected in PanIN and IPMN cells reflect the distinct molecular profiles of these cell types. Further, we hope that these findings will enhance our mechanistic understanding of the characteristic molecular alterations underlying pancreatic cancer precursors. These data may provide a promising direction for therapeutic research.

## Introduction

Pancreatic cancer remains among the most lethal malignancies worldwide, and the incidence of this disease has been increasing slowly in recent years (1–3). The assessments of molecular markers and tumor progression signatures are essential contributors to treatment decisions (4, 5), as a timely diagnosis could prevent 12–13% of patients in a precursor stage or with clinically unapparent disease from progressing toward end-stage disease (6).

To date, three precursors of pancreatic cancer have been identified: pancreatic intraepithelial neoplasia (PanIN; the most common precursor), intraductal papillary mucinous neoplasm (IPMN), and mucinous cystic neoplasm (MCN) (7, 8). PanIN is usually confirmed histologically as it develops into infiltrative adenocarcinoma *via* various steps from low grade to high grade, with gradual morphological changes (9). Early molecular alterations [such as K-ras mutation, epidermal growth factor receptor (EGFR) overexpression, and HER2/neu overexpression] and later events (p16, p53, DPC4, and BRCA inactivation) have been reported to contribute to malignant transformation (10). Animal models of pancreatic cancer have been developed to reproduce and study these benchmark genetic alterations and further our understanding of the underlying mechanisms (11).

One previously described mouse model of pancreatic cancer was developed by the concomitant expression of oncogenic mutant K-ras with a loss of Brg1 or p53 (12). The former model developed cystic neoplastic lesions consistent with human IPMN, whereas the latter developed PanIN similar to the corresponding human condition. Therefore, these murine PanIN and IPMN lesions can be used to generate transcriptome signatures representative of overall pancreatic cancer characteristics.

Advances in next-generation sequencing technologies such as cap analysis of gene expression (CAGE) have led to a comprehensive understanding of the regulatory processes applied to transcribed regions of the genome and the construction of an integrated overview of the transcriptome (13). Particularly, CAGE was originally used to construct a precise map of transcription start sites (TSSs) and elucidate the “promoteromes” of mammalian cells and tissues. In one analysis involving the tagging of m7G caps on mRNAs, nearly 25% of mammalian m7G caps were not located at already known TSS (14, 15). Whole-transcriptome network analyses *via* technologies such as CAGE might enable a much more comprehensive understanding of the divergence of promoteromes in the precursors of malignancies such as pancreatic cancer (16).

Although several mutations have been explicitly identified in pancreatic cancer (17), the role of epigenetic modifications in this malignancy remains largely unknown, and the optimal transcriptional network remains to be discovered. Techniques such as CAGE can be used to evaluate variations in regulatory networks over time and in response to various factors, such as enhancers, mechanical stressors, and stable or long-term regulatory transcriptional factors. Here, in this study, we took a glimpse of the transcriptome network depicted in PanIN and IPMN and hopefully found evidences that might support the future therapy development aiming at the early period of pancreatic cancer.

## Materials and Methods

### Cell Lines

The mouse pancreatic cancer cell lines PanIN and IPMN were cultured in Roswell Park Memorial Institute (RPMI)-1640 medium (Nacalai, Japan) supplemented with 10% heat-inactivated fetal bovine serum (HyClone, USA) and 100 IU penicillin–streptomycin/ml (Nacalai, Japan). The Akt inhibitor GSK690693 (Sigma, USA) and gemcitabine (Tokyo Chemical Industry Co., Japan) were added to the cultures on the second day after seeding. Briefly, the cells were cultured with a fixed concentration of GSK690693 or AZD4547 for the next 4 days.

### RNA Extraction and Isolation

Total RNA was extracted and isolated using the Nucleospin RNA plus kit (Takara, Japan). The concentration and A_260_/A_280_ ratio of each prepared RNA sample were measured using a NanoDrop device (Thermo Fisher Scientific, USA). RNAs with A_260_/A_280_ and 260/230 ratios > 1.7 were subjected to a subsequent analysis. RNA quality was assessed using a Bioanalyzer (Agilent) to ensure an RNA integrity number (RIN) > 7.0.

### Cap Analysis of Gene Expression and Computational Analysis

CAGE library preparation, sequencing, and mapping were performed by DNAFORM (Yokohama, Kanagawa, Japan). CAGE libraries were prepared from the purified RNA samples, as follows: briefly, first-strand cDNAs were transcribed to the 5' ends of capped RNAs and attached to CAGE bar code tags. These tags were sequenced on an Illumina HiSeq 2000 sequencer (Illumina, San Diego, CA, USA) and mapped to the mouse mm9 genome using BWA software (v0.5.9) after discarding ribosomal or non-A/C/G/T base-containing RNAs. For tag clustering, CAGE tag 5' coordinates were input for Reclu clustering (18), with a maximum irreproducible discovery rate of 0.1 and minimum count per million (CPM) of 0.1. The samples were normalized using the relative log expression method, and differential analyses were conducted using edgeR. In the motif analysis, genomic DNA sequences in the regions that are 500 bp upstream to 500 bp downstream of the CAGE peaks were analyzed using the MEME and DREME (Analysis of Motif Enrichment) tool (motif search cutoff: *E* score > 0.05) (19) and the JASPAR CORE 2014 vertebrates 68 motif database. TOMTOM was used for comparisons.

### Quantitative Real-Time PCR

Complementary DNA was synthesized using the Superscript II 1st Strand cDNA synthesis kit (Takara, Japan). Quantitative PCR was performed with the SYBR-green-based gene master (Roche, Germany). The expression levels were normalized against mouse Gapdh (Hokkaido System Science Co., Japan). The primer sequences can be referred in Table S1.

### Western Blotting

Cell lysates (20 μg/lane) were electrophoresed on 6 and 15% commercially purchased gels (SuperSep Ace, Fujifilm, Japan) and then transferred to polyvinylidene difluoride (PVDF) membrane (Immobilon-P, Merck, Ireland). Immunoblotting was performed by primary antibody: anti-RREB1 antibody ab64168 (Abcam, USA), pan Akt C67E7 Rabbit mAb and phosphor-Akt (Thr308) D25E6 Rabbit mAb (CST, USA), and horseradish peroxidase (HRP)-conjugated secondary antibody ab2307391 (Jackson ImmunoResearch, USA). Beta-actin immunoblotting was performed by anti-beta actin antibody ab49900 (Abcam, USA).

### Immunocytochemistry and Immunofluorescence Assay

Cells were seeded on culture slides (Falcon, USA) and fixed with 4% formaldehyde in 1× PBS for 10 min at room temperature. They were then blocked with 1× PBS containing 0.25% Triton X-100 and 5% bovine serum for 60 min at room temperature and incubated with primary antibodies overnight at 4°C: anti-Nanog antibody ab80892 and anti-Sox2 antibody ab92494 (Abcam, USA). On the second day, the cells were washed with 1× PBS and incubated with secondary antibodies for 60 min at room temperature: Alexa Fluor® 488 ab150081 (Abcam, USA). Nuclear staining was observed using DAPI staining (VECTASHIELD, Japan), and images were acquired by Keyence fluorescence microscope.

### Gene Ontology and Kyoto Encyclopedia of Genes and Genomes Pathway Enrichment Analyses

To identify the key pathways associated with differentially expressed promoters, the CAGE data were subjected to a Gene Ontology (GO) enrichment analysis and Kyoto Encyclopedia of Genes and Genomes (KEGG) pathway analysis using the clusterProfiler R package (20). The Goplot package was then used to explore the biological significance of these differences (21). The significant GO terms and pathways were identified using the Benjamini test to generate corrected *P*-values.

### Gene Set Enrichment Analysis

A gene set enrichment analysis (GSEA) was conducted using primarily computational methods to determine the significant differences in expression between PanIN and IPMN from the CAGE datasets. Usually, a false discovery rate (FDR) <0.25 is applied to generate the most likely hypotheses. However, we applied a slightly looser cutoff of 0.5 in this study to accommodate the small number of samples.

### Detection of Aldehyde Dehydrogenase Activity and Flow Cytometry

The ALDEFLUOR kit (StemCell Technologies, USA) was used to identify cells exhibiting aldehyde dehydrogenase (ALDH) activity. Cells were incubated (45 min, 37°C, final densities: 2 × 10^5^ and 5 × 10^5^ cells/ml) with the specific ALDH inhibitor diethylaminobenzaldehyde (DEAB). A fluorescent substrate was added as the background modification. All procedures were conducted according to the provided protocol.

### Sphere Formation Assays

Suspensions of PanIN and IPMN cells were adjusted to a density of 10^4^ cells/ml in medium comprising DMEM/F12 (Nacalai, Japan) supplemented with B27 (Gibco, USA), 100 μg of epidermal growth factor (Thermo, USA), and 50 μg of basic fibroblast growth factor (Thermo, USA). Next, cells were seeded in semi-suspension and cultured on Ultra Low Attachment 6-well plates (Corning Incorporated Life Sciences, USA). After a 5 day incubation, the numbers of cell spheres were counted and recorded using cell^3^imager (SCREEN Holdings, Japan).

### Xenograft Growth in Non-obese Diabetic/Severe Combined Immunodeficiency Mice

All procedures involving animals were conducted in accordance with the Institutional Animal Welfare Guidelines of Kyoto University. Cells (10^6^ per cell line, viability > 95%) were suspended in 80 μl of phosphate-buffered saline (PBS) together with 20 μl of Matrigel and injected subcutaneously (s.c.) into the dorsal regions of 8 week-old non-obese diabetic/severe combined immunodeficiency (NOD/SCID) mice (Charles Rivers Laboratories, Yokohama, Japan; four mice per group). The mice received three subsequent intra-abdominal injections of GSK690693 (Selleck Chemical, Japan) at doses of 40 mg/kg/day. The tumors were harvested after 4 weeks.

### Cell Counting Kit-8 Assay

Cells were seeded in 96-well plates after several days of incubation with the chemotherapy drugs. After the culture media were discarded, the cells were washed with PBS. Ten microliters of Cell Counting Kit-8 (CCK-8; Dojindo, Japan) and 90 μl of culture medium were added to each well. Subsequently, the cells were incubated for 2 h in an atmosphere of 95% air plus 5% CO_2_ at 37°C. A microplate reader (TECAN Infinite F50, USA) was used to detect the optical density value at 450 nm in each well as a measure of cell proliferation.

### Statistical Analysis

R statistical software, version 3.5.3 (http://www.R-project.org), was used to perform the general statistical analysis and generate the graphs. All *P*-values described in this study represent two-sided tests. A *P* < 0.05 was considered statistically significant.

## Results

### Histological and Genome-Wide Assessment of Subcutaneous Pancreatic Intraepithelial Neoplasia-Pancreatic Ductal Adenocarcinoma and Intraductal Papillary Mucinous Neoplasm-Pancreatic Ductal Adenocarcinoma Mouse Cell Tumors

**Figure 1:**
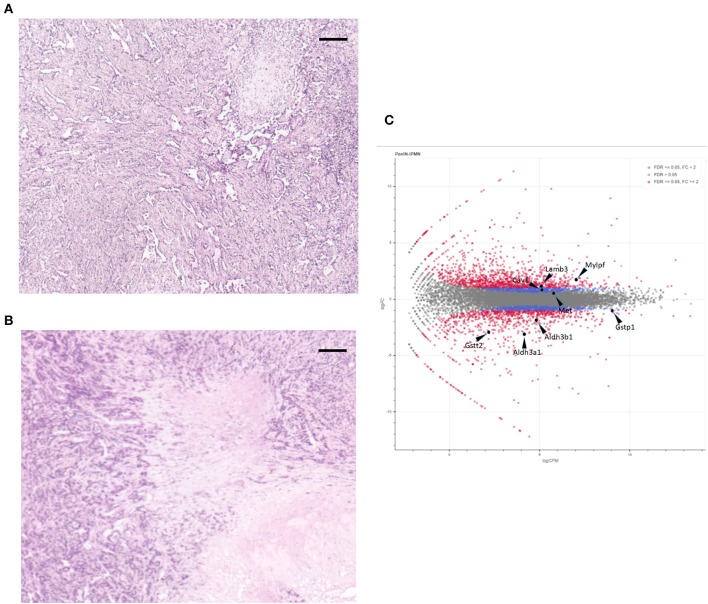
Hematoxylin and eosin staining of subcutaneous tumors formed by cell lines intraductal papillary mucinous neoplasm (IPMN) **(A)** and pancreatic intraepithelial neoplasia (PanIN) **(B)**. Scale bar: 200 μm. **(C)** MA plot of the monitored expressions of individual genes. Non-differentially expressed (gray dots), differentially expressed (red dots), and the eight selected genes are shown.

For these experiments, we used pancreatic cell lines generated from a Kras^+^p53^+^Brg1^−/−^; Kras^+^p53^+^ mouse pancreatic cancer model that was developed by Fukuda et al. After subcutaneous tumors in the NOD/SCID mice had been created *via* injection of these cell lines, hematoxylin and eosin-stained tissue sections were evaluated histologically to confirm that the tumors mimicked pancreatic cancer. As shown in Figures 1A,B, the tumor component contained a clear ductal spectrum consistent with classic pancreatic cancer, with little variation between the two sections, thus verifying little difference in the manifestation of PDA at the advanced stage.

We next applied CAGE to the RNAs extracted from the subcutaneous tumors formed by injecting mouse PanIN and IPMN cell lines into NOD/SCID mice. As shown in Figure S1, a clustering analysis based on the entire genome-wide promoter set revealed clearly distinct trends in the mouse pancreatic cancer cells depending on the PanIN or IPMN status. A MA plot also demonstrated that a differential analysis with an FDR of <5% revealed a balanced distribution of the promoters (Figure 1C).

### Use of Chromatin Immunoprecipitation-Based Weighted Parametric Gene Set Analysis to Create Comprehensive Networks of Transcription Factor Activity

**Figure 2:**
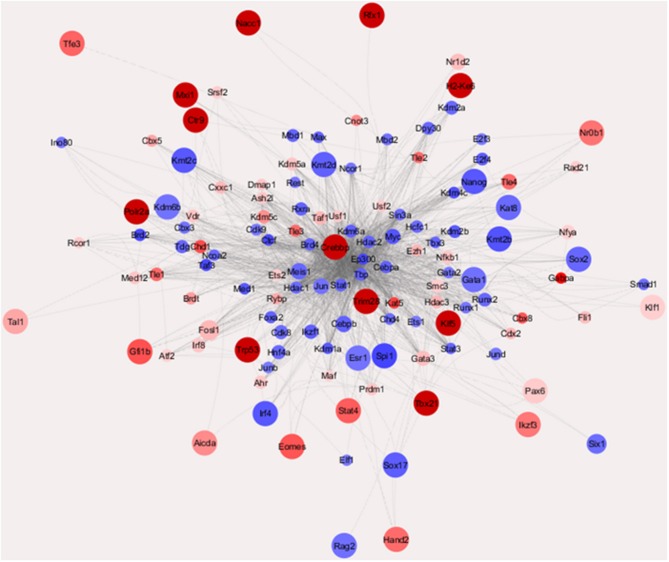
Transcriptional regulatory network of TFs characterizing IPMN and PanIN (Enrichment Score >10 or < −10). Those nodes with more out-degrees [the number of other transcription factor (TFs) that they regulate] were placed in a center position. The node size indicated the LogFC value of the TF gene found in cap analysis of gene expression (CAGE) data and the node colors indicate the enrichment scores of the TFs estimated with weighted parametric gene set analysis (wPGSA).

To get an overall image of the configurations of transcription factor (TF) networks in the two cell lines, we used weighted parametric gene set analysis (wPGSA) to establish a visual network of the factors detected among the differentially expressed promoters (Figure 2) (22), which is a method to estimate relative activities of transcriptional regulators from given transcriptome data. This analysis displayed the dynamic and complex nature of gene regulation in mouse PanIN/IPMN cells, together with the TSS cluster expression profiles. LogFC values were used to represent the relative activities of TFs in the whole PanIN/IPMN transcriptome. Distinct configuration signatures were evident because TFs in the IPMN appeared to be spreading in the outer layer and had less connections than had TFs in the center, of which most were present in PanIN, whereas the number of TFs enriched in the PanIN weighted higher several star molecules, including Nanog, Sox2, and Runx1. These molecules play especially important roles in the epithelial–mesenchymal transition and dedifferentiation processes, indicating a tendency toward aggression at the TF level (23). In IPMN, the transcriptome included classic malignancy-promoting genes such as Stat4, Trp53, and Pax6, suggesting a more rigorous and limited perception of the connections between TFs.

Confirmed with qRT-PCR, which is shown in Figure S2A, most of the star molecules mentioned above had varied mRNA expression in PanIN/IPMN cell lines. Immunocytochemistry (ICC)/immunofluorescence (IF) technique was applied in detecting Nanog and Sox2 expression in PanIN/IPMN (Figures S2B, S2C).

### Identification of the Enriched Processes Among Promoters in Intraductal Papillary Mucinous Neoplasm/Pancreatic Intraepithelial Neoplasia Cells

**Figure 3:**
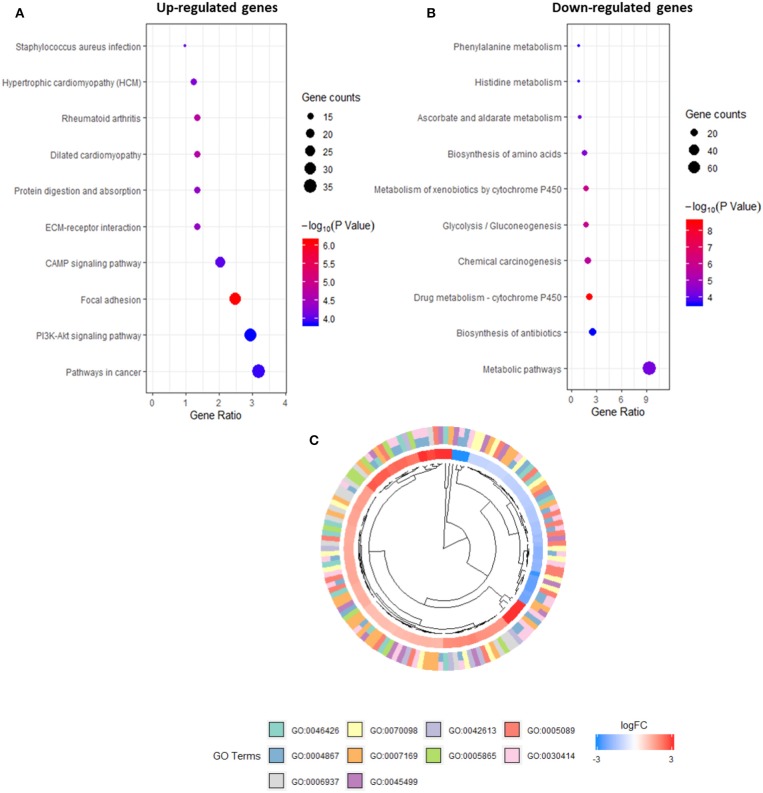
Kyoto Encyclopedia of Genes and Genomes (KEGG) pathway analysis of promoters in up-regulated **(A)** and down-regulated expression **(B)**. Gene Ontology (GO) terms within differentially expressed promoters [false discovery rate (FDR) > 0.05] in intraductal papillary mucinous neoplasm (IPMN) **(C)**.

The promoters identified *via* CAGE were refined further using several criteria: an exact FDR cut-off <0.01 to indicate statistical significance in the differential analysis, a median expression level of >4 CPM to obtain observable results with qRT-PCR and a |LogFC| > 2 to indicate a solid difference in expression between the two groups. A total of 1,261 upregulated and downregulated promoters were screened and tested for enrichment using DAVID to identify those with annotation robust peaks within a cluster associated with a pathway or biological process. A subsequent KEGG/GO term enrichment analysis revealed that the commonly upregulated promoters were enriched primarily in important pathways such as PI3K-Akt signaling and focal adhesion (Figure 3A). The downregulated promoters were enriched in P450 metabolic systems such as drug metabolism and xenobiotic metabolism (Figure 3B). In IPMN cells, the upregulated promoters were enriched for the GO categories related to inhibitory enzymatic activity, negative regulation of cell communication, and immune processes (Figure 3C), indicating a more suppressive signature when compared with that of PanIN cells.

### Distinct Transcription Networks in Mouse Pancreatic Cancer Cell Lines Generated From Pancreatic Intraepithelial Neoplasia-Pancreatic Ductal Adenocarcinoma/Intraductal Papillary Mucinous Neoplasm-Pancreatic Ductal Adenocarcinoma

We also examined the enrichment of TF-binding motifs in the vicinity of the promoters. On the basis of the above pathway analysis results, we focused on promoters associated with the PI3K-Akt signaling pathway, focal adhesion, and P450 metabolism. A further survival analysis based on RNAseq data provided by The Cancer Genome Atlas (TCGA) confirmed that some of these annotated genes exhibited significant correlations with the survival duration in days (*P* < 0.05; Figure S3A). All survival-related genes were then subjected to qRT-PCR (Figure S3B).

**Figure 4:**
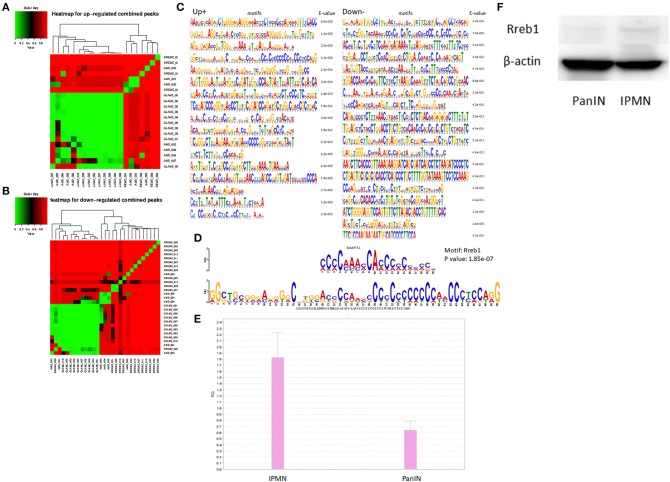
Clusters screened by the differentially expressed criteria and divided into up-regulated or down-regulated peaks were used for motif discovery analysis, heat maps represented results shown as up-regulated and down-regulated in **(A,B)**. **(C)** Motifs significantly detected at the promoter region of target genes (enrichment score < 0.05). **(D)** Comparison analysis with standard motifs representations in the JASPAR core database using TOMTOM. The sequence logos at the top showed the transcription factor MA0073.1 (Rreb1), and the ones below the binding match motif. **(E)** qRT-PCR result suggested Rreb1 expression in pancreatic intraepithelial neoplasia/intraductal papillary mucinous neoplasm (PanIN/IPMN) correlated with findings from motif search. **(F)** Rreb1 protein (245 kDa) expression level of IPMN/PanIN cell lines; beta-actin was used as the internal control.

Next, we applied MEME (24) to the survival-related genes, using several motif search tools. First, we generated heat maps on the basis of the total promoter expression levels to reveal differences in the configurations of various predicted and discovered categories of motifs. The distinct mapping of several sets of motifs is shown in Figures 4A,B. The motifs discovered for promoters associated with regulatory survival-associated genes *via* MEME are shown in Figures 4C,D. These were subjected to a motif comparison using TOMTOM (25), which suggested that the comprehensive transcriptional factor Rreb1 had a significantly upregulated promoter. The binding motif of Rreb1 was also enriched among the upregulated promoters. As shown in Figures 4E,F, qRT-PCR and western blotting verified that the expression of Rreb1 was higher than in the IPMN compared with PanIN. These results indicate that in the mouse, IPMN and PanIN are likely controlled by distinct transcriptional regulatory networks. Similar candidates of transcriptional factors were discovered *via* Motif Activity Response Analysis (26) (MARA; Table S1), which is another classical approach in motif analysis adopted in processing CAGE data.

### Gene Set Enrichment Analysis of the Promoteromes in Intraductal Papillary Mucinous Neoplasm/Pancreatic Intraepithelial Neoplasia Cells

**Figure 5:**
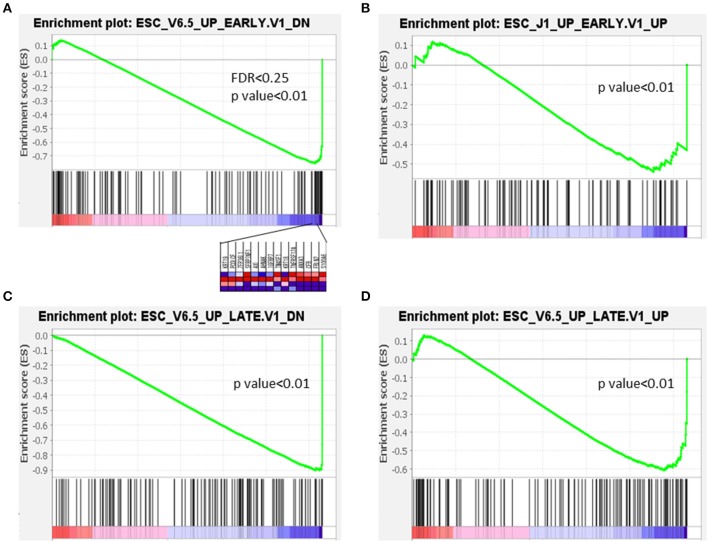
Expression in PanIN derived from mouse PDA was negatively correlated with differentiation of embryoid bodies from mouse embryonic stem cells.

The characteristics of several GSEA datasets from different categories were analyzed using the PanIN/IPMN leading gene CPM. Among all tested datasets, genes that were upregulated and downregulated during the early or late stages of embryoid body differentiation from embryonic stem cells (ESCs) consistently yielded significant differences. As shown in Figure 5, only the expression matrix from PanIN had a detectably distinct pattern different from that of stepwise development in a mouse embryo. On the contrary, PanIN cells exhibited negative correlations with all genes that were downregulated during the early/late differentiation of embryoid bodies from ESCs, suggesting that these cells tend to lose the capacity for pluripotency. PanIN cells also exhibited negative correlations with genes that were upregulated during the early/late differentiation of embryoid bodies. Like other well-differentiated cells, PanIN manifested alliance toward the less oncogenic property of the upregulation of stem signature from early/late stage of ESC. All but the first GSEA result lacked FDR power and were considered marginal. We note, however, that the GSEA datasets corresponding to early/late shifts in ESC genes represent the loss of stemness properties in PanIN. Therefore, these results might suggest the presence of stemness before the transition from PanIN into a solid tumor.

### Validation of Stemness in a Mouse Cell Line Derived From Pancreatic Intraepithelial Neoplasia-Pancreatic Ductal Adenocarcinoma

**Figure 6:**
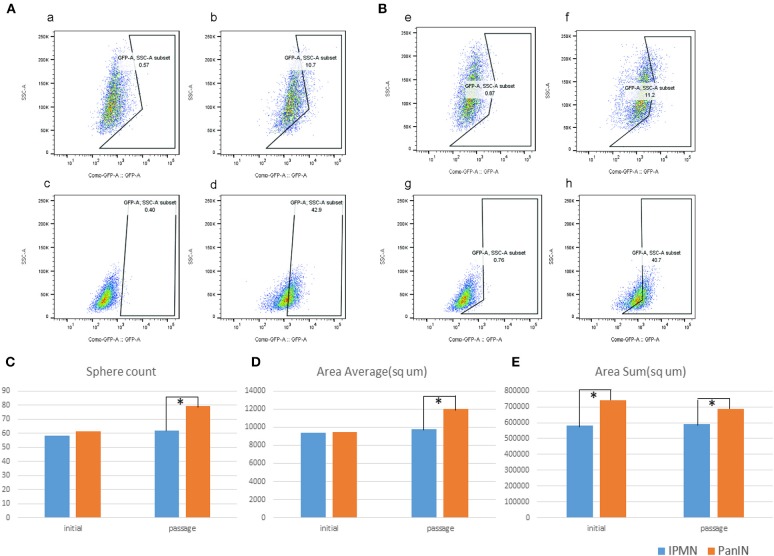
Green fluorescent protein (GAP)-conjugated ALDH^+^ and DAPI (detection of dead cells) were detected by flow cytometry; right, the fraction of ALDH^+^ cells (GFP^+^, DAPI negative) (b,d,f,h) vs. left, as the background correction. Upper were cells from intraductal papillary mucinous neoplasm (IPMN); lower were cells from pancreatic intraepithelial neoplasia (PanIN). In separate condition of 5 × 10^5^ cells/ml **(A)** and 2 × 10^5^ cells/ml. **(B)** Sphere digitalization by cell^3^imager. Sphere count **(C)**, area average/μm^2^ **(D)**, and area sum/μm^2^ **(E)**.

We investigated stemness properties in PanIN- and IPMN-derived mouse PDA cell lines in terms of higher Aldh enzyme activity, as previously reported (27). The Aldh family, which includes Aldh1a1 and Aldh1a3, comprises well-known stem cell markers (28). Therefore, the proportion of Aldh^+^-positive cells after treatment with the Aldh family inhibitor DEAB can be determined by flow cytometry as an indicator of stem cells in various cell types. In this respect, we used the ALDEFLUOR kit (see Materials and Methods) to detect cell populations with higher Aldh activity in both PanIN and IPMN cells *via* flow cytometry. DEAB was used to correct the background fluorescence. Figure 6 demonstrates a significantly higher percentage of Aldh^+^ cells in PanIN than in IPMN (~40% vs. 10%; Figure 6Aa–d) at a cell density of 5 × 10^5^ cells/ml and incubation time of 45 min. Similar results were obtained with a cell density of 2 × 10^5^ cells/ml (Figure 6Be–h).

We next performed a sphere formation assay to assess the stemness property (29). The spheres formed by IPMN cells tended to merge upon reaching a diameter of 100 μm, followed by a transformation into an irregular shape and loss of the round shape (Figures 6C–E, Figure S4). In a comparison of the cell lines, both the numbers of spheres with diameters > 80 μm and the average area of a single sphere were likely to exhibit insignificant differences. After passage, however, PanIN exhibited a greater capacity in terms of overall sphere formation and spherical size. The varied sphere densities and diameters despite culture under the same circumstances revealed the diverse stemness properties of PanIN and IPMN cells.

### Evaluation of Chemoresistance in Intraductal Papillary Mucinous Neoplasm/Pancreatic Intraepithelial Neoplasia-Pancreatic Ductal Adenocarcinoma Cells

**Figure 7:**
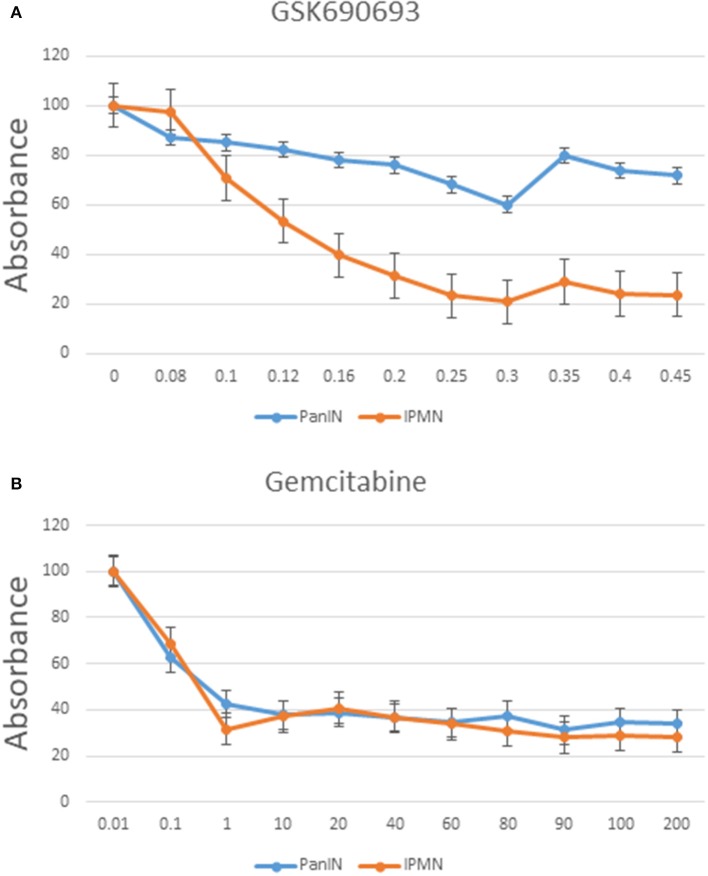
Cell proliferation assay of PanIN and IPMN cells after adding GSK690693 (Akt inhibitor) **(A)** and gemcitabine **(B)** normalized to relative control. Data are shown as mean ± SD performed in triplicate, representative of three independent experiments. *Y* axis: %; *x* axis: μM.

Chemoresistance is a typical characteristic of stemness-possessing cells (30). As mentioned previously, our CAGE data suggested activation of the PI3K-Akt signaling pathway in IPMN-PDA cells. To evaluate this possibility, GSK690693 (Akt inhibitor) was added to cultures of both cell lines, and the effect was investigated using a 5 day CCK-8 cell proliferation assay. Notably, GSK690693 decreased the proliferation rate of IPMN cells in an approximately dose-dependent manner, with a significant decrease in viability at a concentration of 0.3 μM relative to the inhibitor-free control (*P* < 0.05; Figure 7A). PanIN exhibited a relatively insignificant decrease proliferation in response to the same concentration of GSK690693.

This observation prompted us to explore whether GSK690693 would have a similarly anti-proliferative effect on subcutaneous tumors *in vivo* in NOD/SCID mice. GSK690693 was injected intraperitoneally into two random mice from both the PanIN-PDA and IPMN-PDA groups (30 mg/kg/day at three intermittent times). Two untreated mice from each group were also observed. As shown in Figure S3, the sizes of the tumors varied between the two cell lines. However, in the same PanIN/IPMN group, the longitudinal size measured did not differ significantly between GSK690693-treated and untreated mice (Figure S5).

We also treated both cell lines with gemcitabine in accordance with the standard treatment regimen for pancreatic cancer. As shown in Figure 7B, IPMN and PanIN did not differ greatly in terms of sensitivity on gemcitabine; although PanIN had a more rapid proliferation speed in normal culture condition (Figure S6A), gemcitabine inhibitory effect seemed nearly equal when calculated proportionally, indicating a rigorous Akt inhibitory resistance in PanIN alone. Function of Akt inhibitor was verified through western blotting using phospho-Akt and pan AKT (Figure S6B): phosphor-Akt level in IPMN treated with GSK690693 was lower than non-Akti-treated control. Besides, application of another AKT inhibitor, AZD5363, was found generating similar proliferation alteration in PanIN/IPMN (Figure S6C).

Upon Akti treatment, alteration in stem cell contents occurred as well: as can be seen in Figure S7, when cell density reached 10^6^ cells/ml under 45 min of incubation time, a greater drop of Aldh^+^ cells in Akti-treated IPMN than in PanIN can be observed (Figure S7A; *P* < 0.05). The same scenario happened in CD24/44 stem cell system in PanIN/IPMN (Figures S7B, S7C) (31), which was reported as pancreatic cancer stem cell (CSC) markers, CD24/CD44 population decreased significantly in IPMN than in PanIN when treated with Akti.

## Discussion

The transition of a pancreatic cancer precursor into the most lethal malignancy has long been a topic of interest (32, 33). However, the detection of asymptomatic precursor lesions and early-stage disease has been challenging because of a lack of sufficiently sensitive methodology and a poor understanding of these entities (34). Therefore, a practical and accurate mouse model that could be used to assess ongoing molecular alterations in cancer might further relevant experimentation. In this study, we used mouse cancer cell lines generated from mouse models of PanIN (Ptf1a-Cre; Kras^G12D^; p53^f/+^) and IPMN (Ptf1a-Cre; Kras^G12D^; Brg1^f/f^).

Our genome-wide enrichment analysis of all differentially expressed promoters identified enrichment in several pathways, including the PI3K-Akt signaling pathway, which is well-known in the field of cancer and has been targeted using several combined therapeutic approaches (35). This finding suggests that inhibitors targeting the PI3K pathway are promising in terms of therapeutics (36). We also observed enrichment for focal adhesion, which is often mentioned in terms of physiological and disease cellular function remodeling. Notably, the activity of focal adhesion kinase is upregulated in malignancies (37). In our analysis of promoter profiles, cytochrome P450 metabolism was the only enriched pathway in PanIN that caught our attention. Enzymes associated with cytochrome P450 metabolism have been recognized in a wide variety of tumors, and these discoveries might lead to novel treatment options and lifesaving opportunities (38). On the one hand, our KEGG pathway analysis revealed mutual enrichment of pathways at the promoter level and the accumulation of common oncogenic processes. On the other hand, our analysis of classic GO terms revealed the enrichment of suppressive biological processes, cellular components, and molecular functions associated with enzyme inhibition and the negative regulation of cell communication in IPMN cells. This enrichment profile differed significantly from that of PanIN cells and might explain the more favorable outcome associated with IPMN. However, IPMN also tends to proliferate at a relatively slow rate and is less aggressive than other precancerous lesions.

We also evaluated differences in the transcriptional network between PanIN and IPMN cells using TFs screened *via* a chromatin immunoprecipitation (ChIP)-based wPGSA. This type of analysis predicts transcription regulators based on transcriptome and global ChIP data *via* computational approaches. Accordingly, we identified different compositions of key regulators in PanIN and IPMN cells. TFs associated with reprogramming, such as Myc, Sox2, and Nanog, exhibited high activity levels in PanIN cells and targeted considerably activated TFs in PanIN. Another classic TF discovery method involves searching for TF bonds with conventional motifs that are found to be cohering in the specified region of the promoters (39). In the promoter levels of Cav1, Met, Mylpf, and Lamb3, which were all survival significant factors in pancreatic cancer, TF Rreb1 was discovered to be in the minor view. Rreb1 plays a key role in Ras-mediated cell differentiation by upregulating the expression of calcitonin. However, it is not necessarily involved in the proliferation of malignant cells (40). A previous study reported that the RREB1/ZIP3/ZINC complex arises in early-stage human pancreatic cancers and suppresses the development of malignant cells (41). Therefore, the loss of Rreb1 activity may contribute to the proliferative ability of PanIN. Overall, wPGSA solves problems in TFs search from routine motif analysis such as prediction of actual frequencies of bindings between TFs and motifs and overcomes the limitation of the fewer number of motifs already known.

GSEA is another bioinformatics method used to determine an expression signature using a previously published set of relatively well-defined genes (42). In this study, the CSC marker list was downloaded from a previously published list of ESCs. The most negatively related genes included KRT19, ZFP36L1, AHNAK, IGFBP7, and TM4SF1; interestingly, KRT19, AHNAK2, and TM4SF1 were previously reported as promising diagnostic candidates and were upregulated in PDA-promoting malignancy (43–45). In a survival analysis, the overexpression of ZFP36L2 and IGFBP7 was found to be correlated with poor survival in patients with PDA (46, 47). Separately, all these genes indicate carcinogenesis, whereas the combined signature suggests a loss of stemness that corresponded with the significantly higher proportion of Aldh^+^ PanIN cells relative to IPMN cells.

Aldh^+^ and CD cell surface markers are used to identify CSCs *via* two different evaluation systems (48). In a previous report, pancreatic cancer cells expressing CD24/CD44 or CD133 functioned as CSCs and exhibited tumorigenesis and chemoresistance (31). The Aldh^+^ system used in this study demonstrated that PanIN cells contained a larger proportion of cells with stemness. Although the ALDEFLUOR kit used in this study mainly detects Aldh1a1, Aldh1a3, and Aldh2 activity while neglecting that of Aldh3a1 and Aldh3b1 (49), we observed the upregulated expression of several Aldh family members in PanIN cells, which suggests a capacity for aggressive proliferation. Our subsequent sphere formation assay confirmed the stemness of PanIN cells. In an actual 2D culture context, PanIN cells exhibited much more accelerated growth than did IPMN cells, and this vigorous proliferation ability was not altered by treatment with GSK690693, a pan-Akt kinase inhibitor proven effective against pancreatic cancer in various preclinical and clinical trials. Our observation that PanIN and IPMN cells exhibited different sensitivities to the same concentration of GSK690693 suggests that this difference underlies the poor treatment effects of single chemo-drug regimens.

Finally, in our use of CAGE data from PanIN and IPMN cells, while dealing with sequencing data (50), variability is of significant importance and is reflected in multiple steps before and during routine analysis; in the preparation stage, human resources tend to generate greater variability than do congenic mouse strains. Besides, biological variance, like sample collection and time points, can induce many variances, which differ obviously in time course study of a phenotypic phenomenon. In this sense, only increasing the number of replicates will be able to aid in drawing a confident conclusion from the data. In our study, tumor formed *via* mouse cancer cells is the end-stage event, and clustering indicated well-represented consistency between replicates, thus indicating trustworthiness for subsequent experiments. As to the TSS level from the CAGE data, reported by Omori et al. using the mutation profiles (51), it was revealed that only the TSS expression levels shifted between the groups. The lack of newly developed TSS peaks and variants suggested that the genomic profiles of PanIN and IPMN were identical to those of PDA. The independent inclusion of p53 and Brg1 mutations in the mouse genome led to the generation of totally different expression networks in PanIN and IPMN. These differences manifested as distinct phenotypes in the transition from precancerous lesions to pancreatic cancer. The clarification of the major events and characteristics associated with these processes might enable future efforts to prevent the transition to pancreatic malignancy in the future.

## Data Availability Statement

The dataset for this study can be found in the GSE139648 (https://www.ncbi.nlm.nih.gov/geo/query/acc.cgi?acc=GSE139648).

## Ethics Statement

The animal study was reviewed and approved by ARC-MedKyotoUniv (Animal Research Committee, Graduate School of Medicine, Kyoto University).

## Author Contributions

RC and AS contributed to the conception and design of the study. AF provided the mice and worked together with SY in the maintenance of the mice collection. RC performed the statistical analysis and wrote the first draft of the manuscript. MS, NK, HS, and ST corrected sections of the manuscript. All authors contributed to manuscript revision and read and approved the submitted version.

### Conflict of Interest

The authors declare that the research was conducted in the absence of any commercial or financial relationships that could be construed as a potential conflict of interest.

## Supplementary Material

Figure S1 Clustering analysis based on expression of the entire promoter set.

Figure S2 Confirmation of transcriptional factors from wPAGA in PanIN/IPMN. **(A)** qRT-PCR results of all the six TF genes in PanIN/IPMN. Intracellular localization of Nanog in IPMN **(B)** and Sox2 in PanIN **(C)** via immunoflourscence staining. Scale bar: 50 μm.

Figure S3 Verification of eight selected genes using qRT-PCR. **(A)** Survival analysis of eight selected genes from up- and down-regulated promoterome. **(B)** qRT-PCR results of all the eight selected genes from motif search.

Figure S4 Morphology of cells in IPMN **(A)** and PanIN **(B)**. Scale bar: 50 μm.

Figure S5 Subcutaneous tumors harvested after GSK690693 applied in treatment in NOD/SCID mice. Within **(A)** tumors formed in the treated group ① and ② vs. untreated group ③ and ④ in week 3 (*p* < 0.05). **(B,C)** represented the diameter measured of each four mice in week 1 and week 2 (*p* < 0.05).

Figure S6 Cell proliferation assay of PanIN and IPMN cells **(A)** and after AZD5363 (Akt inhibitor) treatment **(C)**. **(B)** Western blotting of phosphor-Akt and pan-Akt expression in PanIN and IPMN cells before and after Akt inhibitor treatment.

Figure S7 FACS analysis of cancer stem cell contents alteration upon adding Akt inhibitor via ALDEFLOUR **(A)** and CD system **(B,C)**.

Table S1 Primer sequences and MARA results.

## Abbreviations

CAGE: Cap analysis of gene expression
CPM: Count per million
ESC: Embryonic stem cell
FDR: False discovery rate
GO: Gene Ontology
GSEA: Gene set enrichment analysis
IPMN: Intraductal papillary mucinous neoplasm
KEGG: Kyoto Encyclopedia of Genes and Genomes
MCN: Mucinous cystic neoplasm
PBS: Phosphate-buffered saline
TSS: Transcription start site
PanIN: Pancreatic intraepithelial neoplasia.
