# Supplementary figures and images for: Promoter-Level Transcriptome Identifies Stemness Associated With Relatively High Proliferation in Pancreatic Cancer Cells
Source: Front Oncol. 2020 Mar 20;10:316. doi: 10.3389/fonc.2020.00316 (PMC7099289; doi:10.3389/fonc.2020.00316)

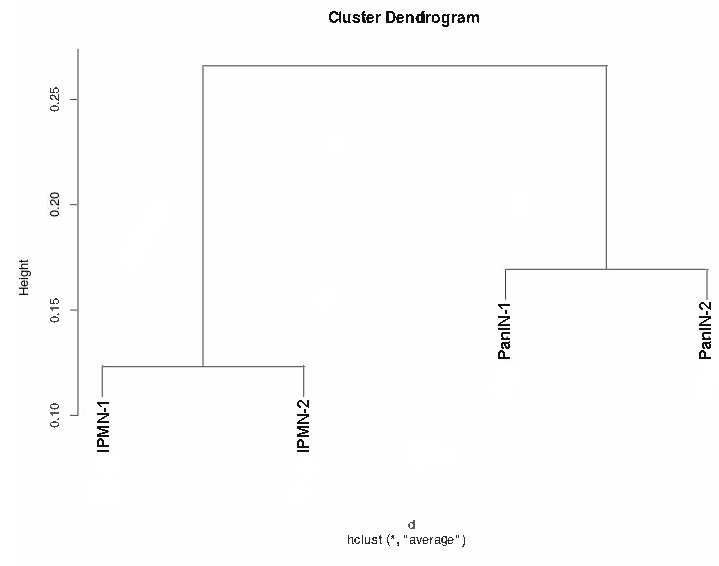

Supplement: Figure S1 — Clustering analysis based on expression of the entire promoter set. [file Image_1.TIF]

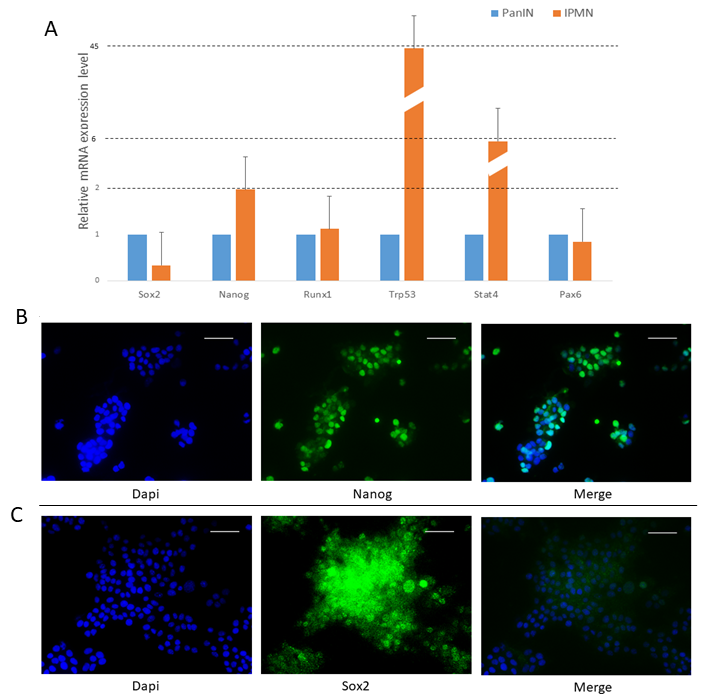

Supplement: Figure S2 — Confirmation of transcriptional factors from wPAGA in PanIN/IPMN. (A) qRT-PCR results of all the six TF genes in PanIN/IPMN. Intracellular localization of Nanog in IPMN (B) and Sox2 in PanIN (C) via immunoflourscence staining. Scale bar: 50 μm. [file Image_2.TIF]

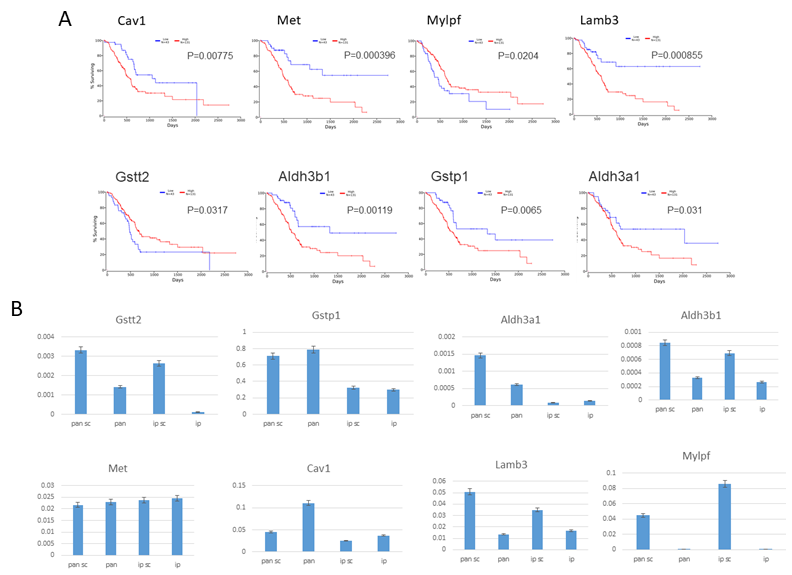

Supplement: Figure S3 — Verification of eight selected genes using qRT-PCR. (A) Survival analysis of eight selected genes from up- and down-regulated promoterome. (B) qRT-PCR results of all the eight selected genes from motif search. [file Image_3.TIF]

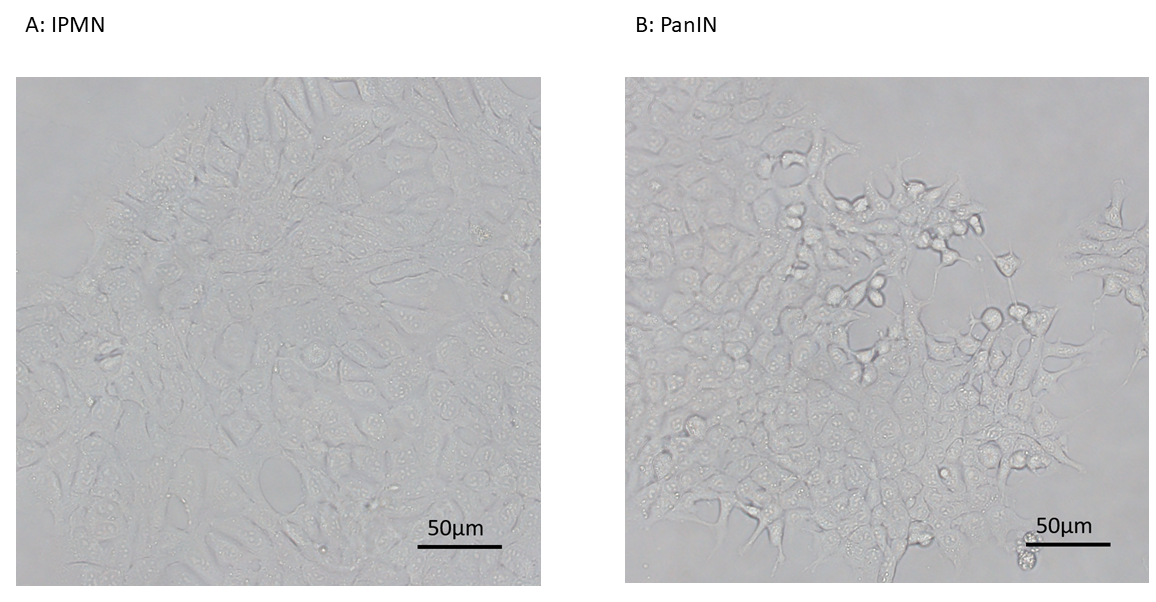

Supplement: Figure S4 — Morphology of cells in IPMN (A) and PanIN (B). Scale bar: 50 μm. [file Image_4.TIF]

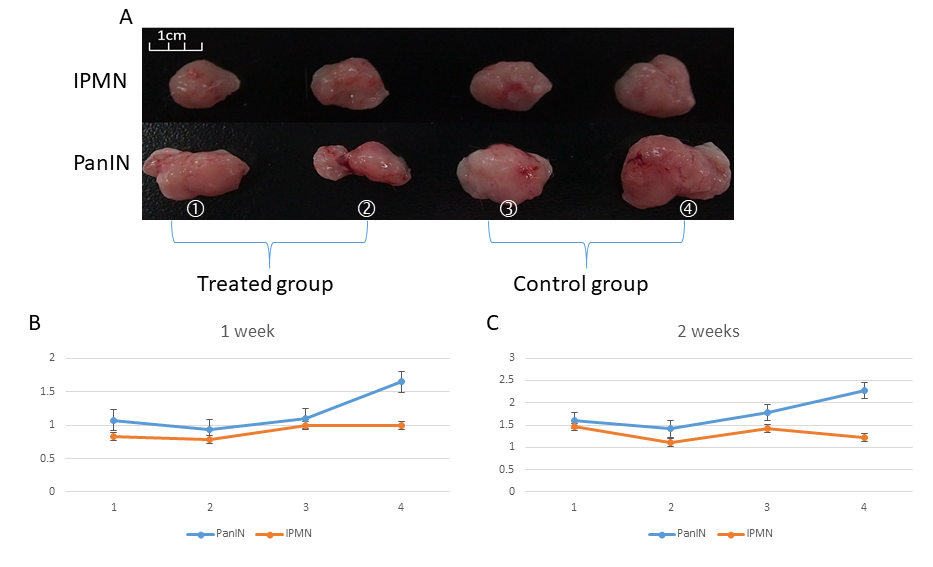

Supplement: Figure S5 — Subcutaneous tumors harvested after GSK690693 applied in treatment in NOD/SCID mice. Within (A) tumors formed in the treated group ① and ② vs. untreated group ③ and ④ in week 3 (p < 0.05). (B,C) represented the diameter measured of each four mice in week 1 and week 2 (p < 0.05). [file Image_5.TIF]

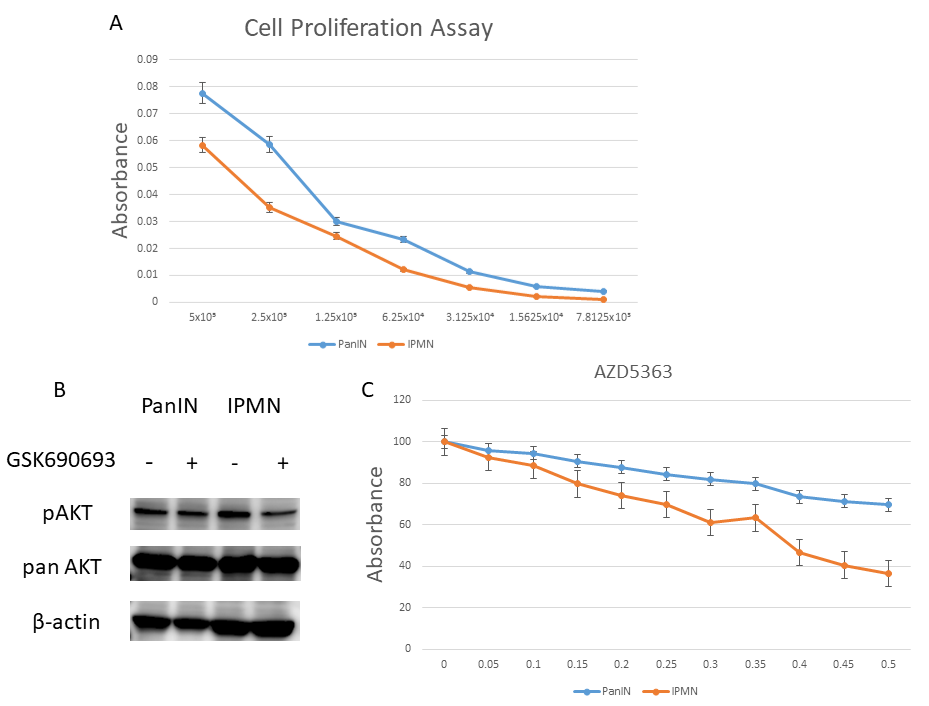

Supplement: Figure S6 — Cell proliferation assay of PanIN and IPMN cells (A) and after AZD5363 (Akt inhibitor) treatment (C). (B) Western blotting of phosphor-Akt and pan-Akt expression in PanIN and IPMN cells before and after Akt inhibitor treatment. [file Image_6.TIF]

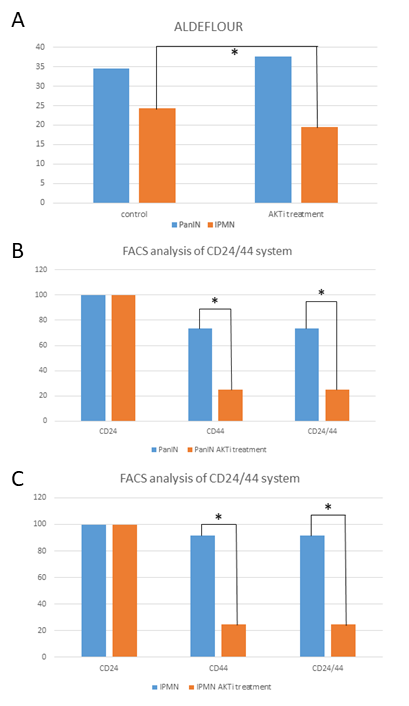

Supplement: Figure S7 — FACS analysis of cancer stem cell contents alteration upon adding Akt inhibitor via ALDEFLOUR (A) and CD system (B,C). [file Image_7.TIF]
